# Mechanism, contributing factors, and coping strategies of alarm fatigue in intensive care nursing: a qualitative study

**DOI:** 10.3389/fpubh.2025.1654389

**Published:** 2025-10-03

**Authors:** Ling Zhu, Siying Wei, Yawen An, Wenjun Hu, Xiaofeng Xie

**Affiliations:** ^1^West China Hospital, Sichuan University/West China School of Nursing, Sichuan University, Chengdu, China; ^2^Innovation Center of Nursing Research, Nursing Key Laboratory of Sichuan Province, Chengdu, China

**Keywords:** alarm fatigue, cognitive load theory, job demands-resources, nurse well-being, patient safety

## Abstract

**Objective:**

To explore the mechanism, contributing factors of alarm fatigue among nurses in Intensive Care Units (ICUs), and to develop targeted coping strategies.

**Methods:**

A combination of purposive and snowball sampling was employed to recruit 27 frontline clinical nurses from various ICU departments. Semi-structured interviews were conducted, and an inductive content analysis of the interview transcripts was performed based on Cognitive Load Theory and the Job Demands-Resources Model.

**Results:**

The study found that alarm fatigue involves dynamic shifts among three cognitive states—cognitive reserve deficit, cognitive load balance, and cognitive overload—with overload being the immediate trigger. Nurses often enter ICU work with limited cognitive reserves. Whether they maintain balance or enter overload depends on the intensity of alarm-related demands and the availability of supportive resources. High-intensity demands for alarm response, such as high alarm frequency, persistent false alarms, multitasking, night shifts, and work–family conflict, are risk factors for alarm fatigue. Resources for alarm response may function as either effective or inadequate support, aligning with protective or risk factors, respectively. Effective support helps alleviate cognitive load and includes effective team collaboration, management’s emphasis on alarm management, comprehensive theoretical training, high psychological adaptability, a strong sense of responsibility, and extensive work experience. Conversely, inadequate support increases cognitive load and includes lack of practical training, absence of formal regulations, outdated and malfunctioning equipment, crowded and noisy layout, emotional personality traits, insufficient or poor sleep, and suboptimal health status.

**Conclusion:**

Cognitive load as a mechanism linking the interaction between alarm response demands and available resources in the development of alarm fatigue among ICU nurses. To mitigate alarm fatigue, it is essential to reduce the intensity of alarm demands while enhancing resource support to relieve cognitive load. Organizational efforts should optimize alarm systems, establish formal protocols, and provide comprehensive training. Teams should reinforce collaboration and mutual support. Individually, nurses are encouraged to enhance psychological self-regulation and maintain sufficient sleep and physical health.

## 1 Introduction

Alarm fatigue refers to the prolonged response time and decreased response rate of clinical healthcare staff to medical device alarms (1), a phenomenon prevalent worldwide. Multiple studies have found that over 60% of alarms do not receive timely responses (2–4), and 85% of nurses report feeling overwhelmed in responding to alarms (5). Studies have shown that alarm fatigue is significantly associated with poorer nursing quality and an increased tendency for medical errors among nurses in Intensive Care Units (ICUs) (6, 7). The Joint Commission reported 98 alarm-related patient injuries between January 2009 and June 2012, including 80 deaths, 13 cases of permanent loss of function, and 5 instances of prolonged care. Notably, the actual incidence of such adverse events may be up to 10 times higher than publicly reported data (8). The United States National Patient Safety Goals have explicitly identified alarm fatigue as a clinical issue for 12 consecutive years (2014–2025), emphasizing the need to “reduce patient harm associated with clinical alarm systems” (9). Technological advancement drives the evolution of clinical practice, and inevitably brings more alarms to intensive care settings (10). Alarm fatigue should not hinder clinical improvements driven by new technologies (11). Furthermore, the COVID-19 pandemic has exacerbated alarm fatigue among ICU nurses (12). World Health Organization (WHO) Director-General Tedros Adhanom Ghebreyesus emphasized that the world will certainly face “the next pandemic” (13). Therefore, addressing alarm fatigue is essential for ensuring patient safety, promoting nurse well-being, implementing innovative technologies, and responding to future public health challenges (14).

Systematic identification and comprehensive understanding of the mechanism and contributing factors of alarm fatigue are essential prerequisites for its scientific and effective intervention. Although factors influencing alarm fatigue among ICU nurses have been investigated, most studies adopt quantitative approaches (15–17). While such studies reveal correlations between factors, they often lack in-depth insight into how these factors affect nurses’ cognitive and emotional responses. Furthermore, findings related to work experience, shift patterns, and training background remain inconsistent (18–20). Although Akturan et al. (12) conducted qualitative interviews addressing related factors, the unique context of the pandemic may have amplified the impact of work environment factors. The fundamental causes of alarm fatigue, along with its risk and protective factors in clinical practice, remain unclear (21). A comprehensive investigation into the causes of alarm fatigue based on ICU nurses’ clinical practice is still lacking.

In fact, ICU alarm management is a task highly reliant on cognitive capacity. These alarms vary in tone, light, and source, each potentially indicating different clinical risks. When managing alarms, nurses must integrate multiple factors, including the patient’s current visible monitoring data and physiological status, condition, medical history, treatment plan, prior nursing experience, and communication with physicians and other healthcare staff (11). Even if no further action is required, the processes of identifying, assessing, and acknowledging alarms significantly increase nurses’ overall workload and cognitive burden (22).

Almost all ICU nurses experience high cognitive load (23). Studies have shown that high cognitive load is closely linked to quality and safety issues, including reduced attentional sensitivity (24), inattentional blindness (25), missed nursing care (26), and occupational injuries (27). The Emergency Care Research Institute (28) reports that alarm misses and related patient harm may result from cognitive overload. Goldart et al. (29) emphasized the need to apply cognitive load theory for a comprehensive understanding of alarm fatigue. Meanwhile, the Job Demands-Resources model has been widely applied in the fields of nursing quality, safety, and occupational health (30, 31). Research indicates that job and personal resources can alleviate cognitive stress (32) and encourage nurses to adopt proactive coping strategies (33). However, a systematic exploration of alarm fatigue grounded in these theoretical models has yet to be adequately conducted.

Therefore, this study integrates cognitive load theory and job demands-resources model to identify the mechanism and factors underlying alarm fatigue, providing a theoretical basis for interventions to improve patient safety and ICU nurses’ well-being.

## 2 Methods

### 2.1 Design

This study adopted a qualitative research approach. In healthcare settings such as the ICU, qualitative research is essential for exploring the perceptual barriers and facilitators of phenomena like alarm fatigue, offering valuable insights into healthcare professionals’ experiences and providing contextual understanding (34). The manuscript followed the Consolidated Criteria for Reporting Qualitative Research (COREQ) guidelines (35).

### 2.2 Theoretical framework

This study was based on cognitive load theory and job demands-resources model. According to cognitive load theory, cognitive structure includes long-term and working memory. Long-term memory stores vast amounts of information, while working memory has limited capacity and duration (36). When new information exceeds working memory capacity, fatigue and decreased performance occur (37). Cognitive load has three dimensions: intrinsic, extraneous, and germane load (38). The sum of intrinsic and extraneous load, minus germane load, determines the nurse’s overall cognitive load. The job demands-resources model explains how job characteristics affect cognitive load, classifying them into job demands and resources, with personal resources also playing a crucial role (32).

**Figure 1:**
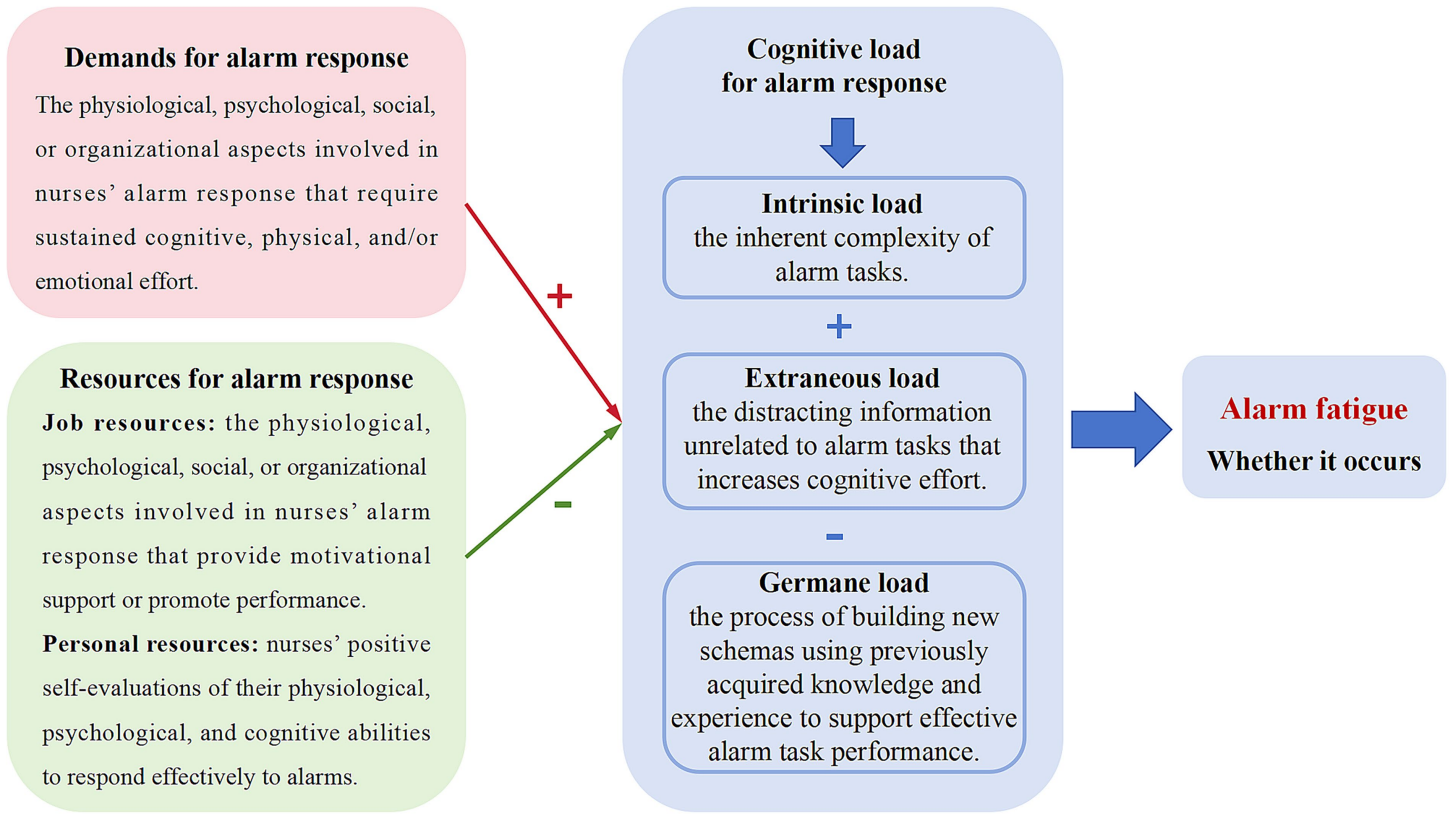
The theoretical framework of alarm fatigue among ICU nurses.

Therefore, the theoretical framework for alarm fatigue is presented in Figure 1. Alarm response tasks typically demand substantial physical, emotional, and cognitive effort from nurses, thereby increasing their cognitive load. Adequate alarm response resources, including both job and personal resources, help alleviate cognitive load. Ultimately, the nurse’s cognitive load state is strongly associated with the onset of alarm fatigue. When alarm-related demands are too high and supportive resources are inadequate, cognitive overload may occur, leading to alarm fatigue.

### 2.3 Setting and participants

Purposive and snowball sampling were used to recruit ICU nurses from three tertiary teaching hospitals in Southwest China. Inclusion criteria were: (1) registered nurses with a valid license; (2) currently working in an ICU; and (3) informed and signed consent. Nurses in internships, further training, or not involved in direct patient care were excluded. To maximize variation, participants of different genders and work experience were recruited from both general and specialized ICUs. The occurrence of alarm fatigue was not required at the time of interview to ensure a comprehensive perspective (39). No financial incentives were provided to avoid potential bias. Data collection continued until saturation was reached, with no new information emerging (40).

### 2.4 Data collection

Semi-structured interviews were conducted face-to-face between January and April 2025. The principal researcher (LZ), a female master’s student skilled in qualitative research methods, conducted the interviews. There was no prior relationship between the researcher and the participants. To ensure a comfortable and non-threatening environment, interviews were scheduled at times and locations convenient for participants, allowing them to share their views freely. Before each interview, the researcher introduced her clinical background and explained the study’s purpose to establish rapport and trust. All interviews were conducted one-on-one by the same researcher, lasted approximately 40 min, and were audio-recorded for subsequent analysis. The interview guide was developed with reference to existing studies (41) and finalized through discussion among the research team (LZ, SYW, YWA, WJH, XFX). It consisted primarily of open-ended questions (Appendix A) and was expanded as the study progressed to explore emerging topics in greater depth. In addition, a study-specific questionnaire was used to gather participants’ basic information. Data collection and analysis were conducted concurrently using the constant comparative method, whereby each interview was compared with previous ones. Data saturation was reached after the 24th interview, and three additional interviews were conducted to confirm saturation (42).

### 2.5 Data analysis

The interview recordings were first transcribed into text using the iFlytek Hearing software. The transcripts were then manually checked for accuracy, with relevant emotional notes added where appropriate. Once verified, the original recordings were deleted to ensure participants’ privacy.

Data were analyzed using an inductive content analysis approach (43), with data management supported by NVivo 15.0 software. Prior to coding, two trained researchers (LZ and SYW) independently read the transcripts multiple times to immerse themselves in the data and develop a comprehensive understanding. During coding, the data were broken down sentence by sentence to identify meaningful statements, which were then open-coded. Coding discrepancies were discussed and resolved through consensus; when necessary, a third expert (XFX) was consulted. Finally, relevant codes were grouped into categories and sub-categories in accordance with the theoretical framework. All analytical results were reviewed and agreed upon by the research team. Examples of the coding process can be found in Table 1 and Appendix B.

**Table 1:** Categories and representative quotes of alarm fatigue.

Theme	Category	Sub-category	Codes	Representative quotes
Mechanism of alarm fatigue	Cognitive reserve deficit	Inappropriate alarm perception	inattentional deafness	“When newly arrived, their sensitivity is relatively low. For example, when something triggers an alarm. they only know there is a sound nearby that keeps going off.” (N24)
			overreaction	“Back when I was new, I would definitely ignore everything else and just focus on dealing with the alarm first.” (N6)
		Difficulty in alarm response	difficulty in identifying alarm sources	“When I first got into the unit, I often found myself looking everywhere to locate which machine was sounding.” (N11)
			Difficulty handling alarms	“At the beginning, I did not understand why the alarm was sounding, nor did I know how to respond to it.” (N1)
		Emotional and psychological burden	nervousness, fear, and anxiety	“When I first started, these feelings were the most obvious—I was worried, nervous, afraid that something might go wrong, and very anxious.” (N27)
			dreaming about alarms	“I even dream about them, which is terrifying. It’s like working two shifts back-to-back and feeling utterly exhausted.” (N18)
			lingering alarm sounds	“When I first started working in the ICU, I would experience that—after getting off work and lying in bed at home, the sounds of monitors, ventilators, and infusion pumps still seemed to surround me, ringing in my ears.” (N24)
			disturbed sleep	“At the beginning, one of our male colleagues could not sleep at all. He’d show up in the morning with seriously bloodshot eyes.” (N23)
	Cognitive load balance	Improved alarm perception	increased alertness	“Once you gain more specialized knowledge, you become more alert—when you hear an alarm, you instinctively check if it’s your patient.” (N21)
		Optimized alarm response	Faster alarm source identification	“Because you are already familiar with the sound, the moment you hear it, you know which machine is alarming.” (N6)
			Faster alarm prioritization	“I can clearly tell the alarm’s priority from its color and tone changes.” (N12)
			Anticipatory action	“I prepare the fluid in advance and replace it just before it runs out, so the pump does not trigger an alarm.” (N1)
			Minimized workflow disruption	“If it’s really urgent, I’ll respond right away. But if it’s not important, I’ll finish what I’m doing first before handling it.” (N6)
		Emotional and psychological adaptation	Calmness	“I do not get annoyed by the alarm sounds because I know it’s just part of the job.” (N4)
			Confidence	“As I’ve gained more knowledge, I’ve become less nervous. Now, most of the time, I can handle things on my own.” (N7)
	Cognitive overload	Impaired alarm perception	Diminished alertness	“After spending a long time in this environment, you kind of become immune to the alarm sounds—you still hear them, but you are not as alert as before.” (N13)
		Inadequate alarm response	Selective ignoring	“Sometimes I feel like I can handle it—if I think the alarm is not that serious, I may not pay much attention.” (N14)
			Simplified judgment	“Some nurses might glance at the alarm, and if it exceeds the normal range but does not affect vital signs, they just let it keep beeping.” (N16)
			Shortened procedure	“For example, when a pump runs out and suddenly alarms, you might find it annoying at night and rush to prepare a new infusion—sometimes skipping steps, which violates standard procedures.” (N6)
			Intentional alarm disabling	“Sometimes I see other nurses turn off the blood pressure monitoring—because once it’s off, the machine will not alarm, even if the blood pressure drops to 20.” (N23)
		Emotional and psychological exhaustion	Irritability	“I get irritated; it makes me feel really uneasy inside.” (N3)
			Alarm numbness	“The device could be alarming right in front of him, and he’d just ignore it—no judgment, no reaction, as if he neither saw nor heard anything.” (N24)
			Auditory intolerance	“The alarm noise at work is constant and overwhelming, so after work, I try to avoid noisy places like supermarkets and bars to fully relax.” (N12)
			Headache	“After work, I often get a headache and just want to find a quiet place.” (N15)
			Sleep disorder	“Many of my colleagues have to take medications like alprazolam to fall asleep.” (N15)

### 2.6 Ethics statement

The study was approved by the Ethics Committee of the relevant hospital (Approval No. 20242621). All participants were informed of the principles of autonomy and confidentiality and provided written consent. Interview data were stored on an encrypted USB drive accessible only to the research team. Each participant was assigned a unique identification number to ensure anonymity. Consent forms and identification records were stored separately in locked file cabinets.

### 2.7 Rigour

The research team received standardized training in qualitative methods and adhered to Lincoln and Guba’s (44) criteria of credibility, transferability, dependability, and confirmability. During the interviews, the researcher recorded participants’ non-verbal behaviors, including facial expressions, body language, and tone, to ensure more comprehensive data. Recordings were transcribed promptly and reviewed line by line for accuracy and to minimize bias. A double-coding process, along with team discussions, further strengthened the study’s credibility, dependability, and confirmability. Detailed descriptions of the study context, participants, and procedures supported transferability.

## 3 Results

A total of 27 ICU nurses were interviewed, drawn from general and specialized units, such as medical, surgical, emergency, cardiac, and neurological ICUs, with varied work experience to ensure sample diversity and representativeness. The characteristics of participants are detailed in Table 2.

**Table 2:** Basic characteristics of participants.

ID	Gender	Age (years)	Education level	ICU type	Work experience (years)	Leadership role
N1	Female	29	MD	MICU	1	No
N2	Male	28	BD	MICU	4	No
N3	Male	31	BD	RICU	8	No
N4	Male	33	BD	MICU	11	No
N5	Female	23	BD	MICU	<1	No
N6	Male	26	MD	SICU	4	No
N7	Male	28	BD	SICU	5	No
N8	Female	24	BD	NICU(neuro)	<1	No
N9	Female	27	BD	SICU	4	No
N10	Female	35	BD	SICU	13	No
N11	Female	24	BD	GICU	<1	No
N12	Male	33	MD	SICU	11	No
N13	Female	27	BD	EICU	1	No
N14	Female	35	BD	GICU	10	Yes
N15	Female	35	BD	GICU	10	Yes
N16	Female	24	BD	PICU	<1	No
N17	Female	30	BD	GICU	5	No
N18	Female	26	BD	GICU	<1	No
N19	Female	33	BD	NICU(neuro)	9	Yes
N20	Female	38	BD	NICU(neuro)	18	Yes
N21	Female	35	BD	TSICU	12	No
N22	Female	42	BD	GICU	22	Yes
N23	Female	32	BD	TSICU	8	No
N24	Female	34	BD	EICU	4	Yes
N25	Female	37	BD	EICU	6	Yes
N26	Female	28	BD	CCU	2	No
N27	Female	28	MD	NICU(neo)	1	No

MD, Master’s degree; BD, Bachelor’s degree; MICU, Medical Intensive Care Unit; RICU, Respiratory Intensive Care Unit; SICU, Surgical Intensive Care Unit; NICU (neuro), Neurological Intensive Care Unit; GICU, General Intensive Care Unit; EICU, Emergency Intensive Care Unit; PICU, Pediatric Intensive Care Unit; TSICU, Thoracic Surgery Intensive Care Unit; CCU, Cardiac Intensive Care Unit; NICU (neo), Neonatal Intensive Care Unit.

Based on the theoretical framework, five categories were identified and organized under two overarching themes: (1) the mechanism of alarm fatigue; and (2) factors contributing to alarm fatigue.

### 3.1 Mechanism of alarm fatigue

**Figure 2:**
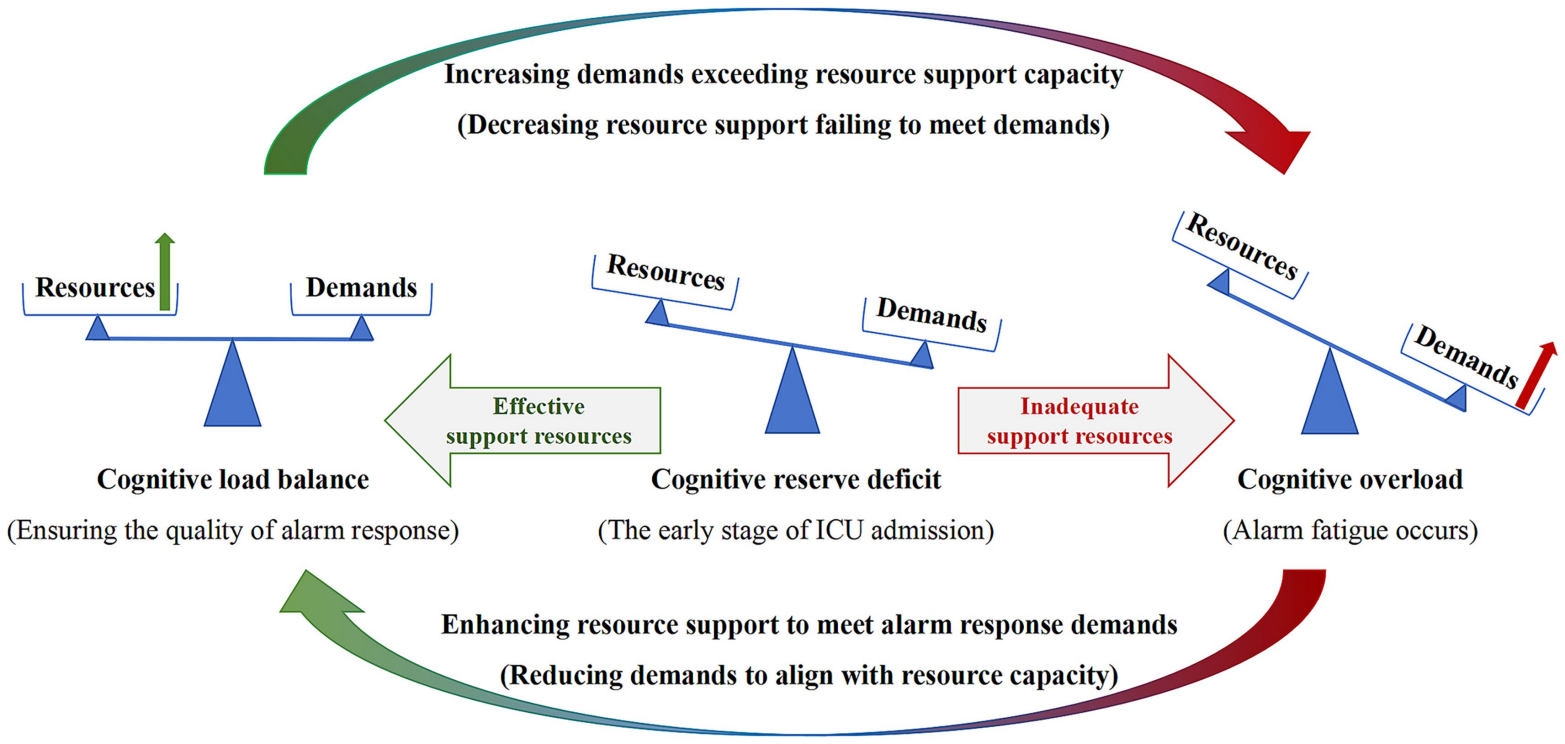
Mechanism of alarm fatigue.

The interview findings suggest that alarm fatigue develops through three states: cognitive reserve deficit, cognitive load balance, and cognitive overload (Figure 2; Table 1). Upon initial entry into the ICU, nurses often experience a mismatch between high alarm response demands and limited cognitive resources, primarily due to insufficient knowledge and experience in critical care, resulting in challenges in alarm management. As clinical experience accumulates and integration into the unit and team improves, support from both job and personal resources strengthens, enabling nurses to meet alarm demands and maintain cognitive load balance. However, when resource support remains inadequate, cognitive overload may occur, serving as a key trigger of alarm fatigue. Importantly, alarm demands and resources fluctuate over time, influenced by elements such as patient conditions, team staffing, and nurses’ physical and psychological status. Thus, cognitive load balance and overload are not fixed states but shift dynamically depending on the interplay between demands and available resources.

#### 3.1.1 Cognitive reserve deficit

Inappropriate alarm perception: At this stage, nurses exhibit relatively weak alarm perception abilities, which may manifest in two ways. First, due to the lack of a developed ability to prioritize alarms, nurses may struggle to recognize the importance of alarms in a timely manner, resulting in alarm insensitivity and even “inattentional deafness” during multitasking. As N21 stated: “A clear phenomenon is that newly assigned nurses may not even realize an alarm is sounding right next to them.” Alternatively, nurses may treat all alarms as equally urgent, leading to “alarm overreaction.” They tend to set aside ongoing tasks in order to respond to alarms. N15 described: “For any alarm, they cannot anticipate what will happen; their immediate thought is that there is an alarm, and they need to address it, so they abandon everything else in their hands.”

Difficulty in alarm response: Due to a lack of sufficient experience and professional knowledge, nurses at the early stage often struggle to quickly and accurately identify the source of alarms. During the response process, they may exhibit confusion and uncertainty, finding it difficult to take appropriate actions promptly. N27 described her early experience: “As for the ventilator and humidifier bottle, you cannot distinguish between them. When you hear the alarm, you wonder what is sounding. You look around for a long time, but you might not find it immediately. You follow the sound and eventually see the device, but you do not understand why it is alarming.”

Emotional and psychological burden: When challenges related to alarm perception, information processing, and coping strategies exceed nurses’ cognitive reserves, they often experience significant emotional and psychological burden, manifesting as nervousness, fear, and anxiety. This stress is not confined to working hours but may extend into off-duty periods, presenting as auditory hallucinations of alarms, recurrent dreams about alarm situations, and decreased sleep quality, thereby impacting their physical and mental health. N27 recalled: “When I first started, these feelings were the most obvious—I was worried, nervous, afraid that something might go wrong, and very anxious.”

#### 3.1.2 Cognitive load balance

Improved alarm perception: With increasing experience, nurses’ sensitivity to alarms improves, and their ability to accurately identify alarms significantly enhances. When an alarm sounds, they can quickly integrate information such as tone, color, and the patient’s clinical status to determine the alarm’s source and urgency without needing to check each device individually. N11 remarked: “Actually, I think the longer you work, the more sensitive you become to alarms (…). you can quickly identify where the alarm is coming from.”

Optimized alarm response: Nurses’ alarm management gradually evolves from mechanical reactions to more anticipatory and thoughtful responses. They can quickly analyze alarm information, consider the patient’s condition, make calm judgments, and flexibly coordinate other nursing tasks with alarm handling. This allows for effective adjustment of response pacing, aligning alarm management more closely with clinical needs and avoiding unnecessary interruptions, thereby improving the continuity and efficiency of overall nursing care. N21 described: “When you are very familiar with your patient and the medications they receive, you can anticipate which medication pump alarm might go off and when. This helps you decide whether immediate action is necessary.”

Emotional and psychological adaptation: Building on effective alarm response skills, nurses gradually internalize alarm management as part of their routine care, no longer dominated by the uncertainty alarms bring. They develop stable psychological expectations, with a significant reduction in emotional tension and anxiety, enabling calmer and more efficient responses to complex alarm situations. N9 shared: “When I first started, I knew nothing about ventilators. For ventilator alarms, I had to call the respiratory therapist. But now it is different. Once your theoretical knowledge improves, you can handle it yourself, so the pressure gradually decreases.”

#### 3.1.3 Cognitive overload

Impaired alarm perception: In environments with prolonged high-frequency alarms, nurses gradually develop maladaptive responses, leading to an increased threshold for alarm perception and contributing to alarm fatigue. “There is fatigue; when you do something for a long time, you become less alert… it’s like human tolerance, where your threshold for alarms increases.” (N23).

Inadequate alarm response: Nurses may exhibit inadequate alarm responses in various forms. These include selective ignoring of low-priority alarms, simplified judgment based on habitual thinking, and shortened procedures that bypass standard steps. In some cases, alarms may even be intentionally disabled. While such actions may conserve physical and cognitive effort, they risk delayed or inappropriate responses, compromising patient safety and quality of care. As N5 noted: “For example, if a patient has been admitted for several days and the same alarm sounds continuously, then I would not check or manage it; I would just press the button to silence it.” A nurse recounted an adverse event involving a colleague: “She had her back to the patient when the patient’s blood pressure suddenly dropped. Because the blood pressure alarm had been silenced, the problem was not detected in time. Although resuscitation was ultimately successful, the patient’s recovery was less than ideal.” (N23).

Emotional and psychological exhaustion: During alarm management, nurses’ physical, cognitive, and emotional resources are continuously depleted, leading to two typical emotional and psychological responses. One group becomes irritable and impatient with environmental stimuli, particularly alarm sounds. As N5 stated: “I get really annoyed whenever the alarm goes off.” Some nurses reported deliberately avoiding noisy environments after work to relieve alarm fatigue. N12 explained: “The alarm noise at work is constant and overwhelming, so after work, I try to avoid noisy places like supermarkets and bars to fully relax.” The other group gradually enters a state of emotional numbness, showing dulled responses to alarms and even passive coping attitudes. N14 noted, “Some senior nurses may feel that these alarms do not really threaten them, so they do not care and just let it go.” Additionally, some nurses mentioned that cognitive exhaustion is accompanied by physical and psychological symptoms, including headaches, sleep disorders, and even dependence on sleep aids. N15 shared: “After work, I often get a headache and just want to find a quiet place (…). Many of my colleagues have to take medications like alprazolam to fall asleep.”

### 3.2 Factors contributing to alarm fatigue

**Figure 3:**
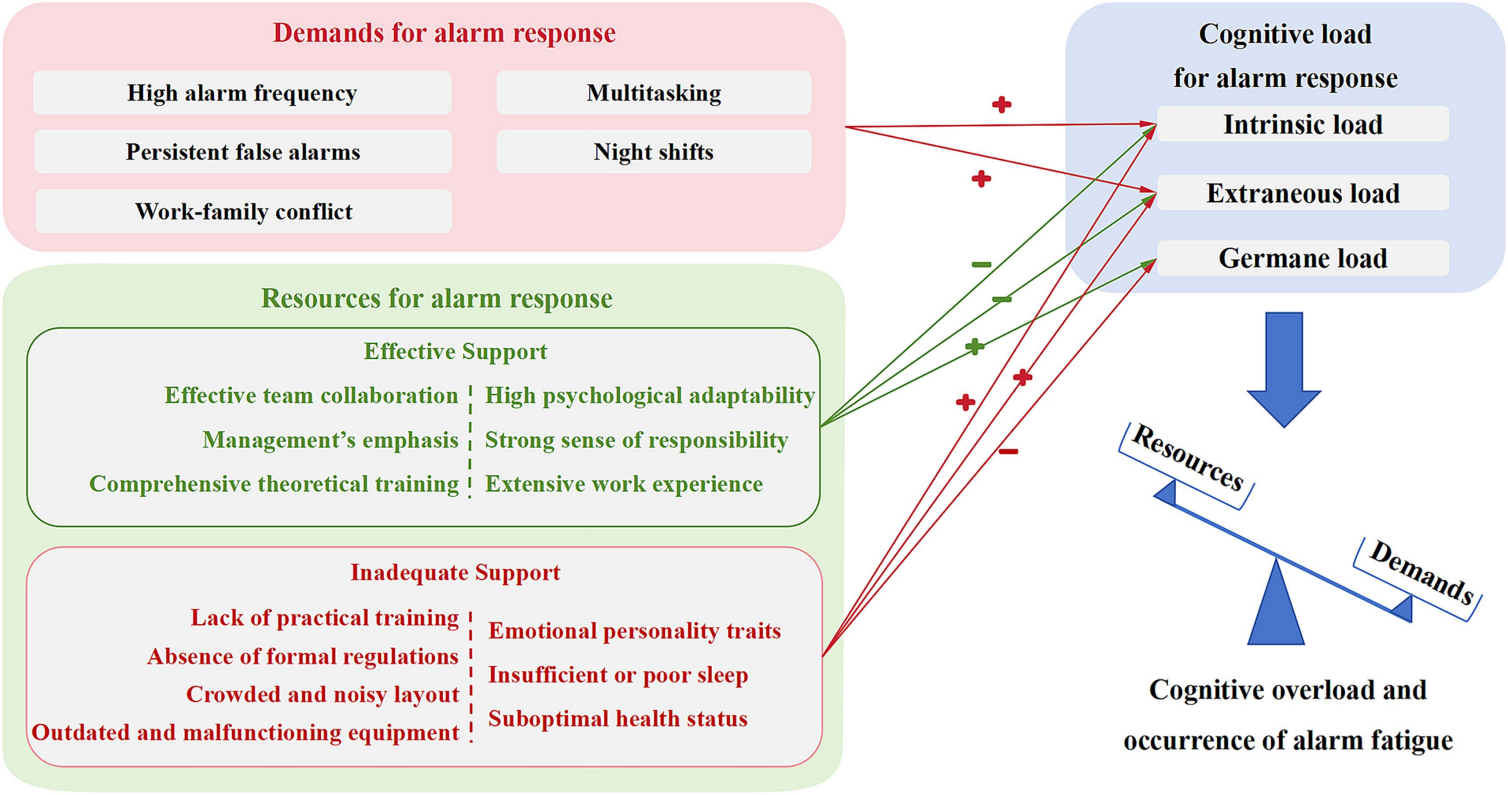
An integrated framework of alarm fatigue in ICU nurses. Arrows marked “+” indicate factors that increase the corresponding type of cognitive load; arrows marked “–” indicate factors that help mitigate it. Items on the left side of the vertical dashed line represent job resources, and items on the right represent personal resources.

Based on open coding, cognitive load theory and the Job Demands-Resources model are applied to structure the factors influencing alarm fatigue. These factors fall into two domains: high-intensity demands, which increase nurses’ cognitive load, and multilevel resources, which provide support in alarm management. Resources are further classified as supportive or inadequate, and differentiated by source into job-related and personal resources (Figure 3).

#### 3.2.1 High-intensity demands for alarm response

High alarm frequency: Some nurses consider high alarm frequency a direct cause of alarm fatigue. As N14 stated: “I think alarm fatigue is quite common now because alarms are going off everywhere in the ICU. There are just too many alarms.” The number of alarms is primarily influenced by the stability of patients’ conditions, exhibiting clear dynamic variability and a degree of uncontrollability. N11 reflected: “It really depends on the patient’s condition. When it’s unstable, there are many alarms; when it’s stable, there are far fewer.”

Persistent false alarms: Persistent false alarms lead nurses to develop a “cry wolf” effect, resulting in delayed alarm responses. As N17 mentioned: “After hearing it so many times, you start to think it’s false, so you do not pay attention and respond more slowly.” False alarms are influenced not only by device sensitivity but also closely related to patient cooperation. N21 noted: “Sometimes patients are uncooperative, causing electrode detachment or inaccurate arterial blood pressure measurements, which trigger false alarms and make us feel frustrated.”

Multitasking: In fact, alarm fatigue is not only caused by alarms themselves but is also significantly influenced by the combined load imposed by multitasking. Frequent alarms increase conflicts with other nursing duties, thereby elevating nurses’ cognitive load and causing them to feel overwhelmed when responding to alarms. As N14 admitted: “When you are busy and an alarm goes off at the same time, one pair of hands cannot handle so many tasks, which leads to irritation and anxiety.”

Night shifts: Nurses commonly report feeling more fatigued and drowsy during night shifts, leading to decreased efficiency in alarm management. N22 described: “I feel completely exhausted; both mentally and physically, I’m not at my best, and my reactions aren’t as quick as during day shifts.” Alarm sounds at night are often perceived as more piercing, which can easily trigger irritability. N12 mentioned: “During the late night shift, we get up at 2 a.m., and our biological rhythms are completely disrupted. At that time, alarms seem especially piercing or even exaggerated. It makes me feel very irritated.”

Work–family conflict: Nurses agree that when family members are ill or emergencies arise, their attention often shifts toward family matters, impacting their ability to manage alarms. As N12 stated: “Because you are worrying, it greatly reduces the attention you have to handle alarms.” This highlights how family responsibilities affect nurses’ emotions and focus, leading to delayed responses.

#### 3.2.2 Multi-level resources for alarm response

##### 3.2.2.1 Effective support resources for alarm response

Effective team collaboration: Effective team collaboration can significantly help share the workload among nurses, alleviating the pressure caused by high-intensity job demands and thereby improving alarm response efficiency. N6 explained: “If you are overwhelmed, you can call the team leader or other less busy colleagues to help. Then, you can better manage alarms, gain more time, and reduce your workload.” Beyond workload sharing, teamwork also facilitates mutual monitoring of alarm handling and helps prevent adverse events. As N15 noted: “Level-1 alarms are extremely sharp and piercing. If you do not respond, others will hear it too. They’ll worry no one is handling it and come to check.” This illustrates a situational form of proactive save, in which team members, though not formally assigned, remain alert to unattended alarms and step in to ensure timely responses and reduce potential risks. Meanwhile, a positive team atmosphere helps relieve emotional stress. N4 mentioned: “Because there are more people during the day shift, I feel a sense of security knowing someone is there to help me.”

Management’s emphasis: Nurses commonly believe that management’s emphasis on alarm management and their expectations encourage timely alarm responses. As N16 stated: “Our team leader requires that when an alarm sounds, we must check it; the alarm cannot be allowed to keep ringing.” Moreover, management’s feedback and communication help maintain high-quality alarm management. A nursing team leader (N22) reported: “Because nursing work is tedious, if reminders are not given regularly, complacency may set in.” Beyond influencing behavioral responses, managerial attitudes also affect the updating and allocation of hardware resources within units. N8 complained: “Our unit has one of the oldest green pumps, which is very inefficient. But it has never been replaced. If you want to replace it, would you pay out of your own pocket for a new pump?”

Comprehensive theoretical training: Training helps nurses better understand and operate medical equipment, particularly alarm response, thereby enhancing their cognitive reserves. Nurses generally acknowledge that the organization provides systematic training on equipment use, alarm management, and emergency protocols. As N9 noted: “In our department’s training, we learned how to recognize alarm color codes, common alarm causes and responses.” N22 confirmed: “Training definitely helps improve the cognitive level of newly hired nurses.”

Strong psychological adaptability: Most nurses believe that individual psychological adaptability is crucial when facing continuously sounding alarms. N4 mentioned: “I have to get used to it. The ICU will definitely have various alarm sounds, and this is unavoidable.” N16 added: “I have to adjust my mindset because I’m responsible for the patient’s life. So even if the alarm sounds repeatedly, my first reaction must be to respond.”

Strong sense of responsibility: A strong sense of responsibility drives nurses to respond promptly to alarms, ensuring patient safety. N6 stated: “As a nurse, I should address alarms as early and quickly as possible.” N2 emphasized: “If I do not, it could endanger the patient’s life.” In addition, strong responsibility motivates nurses to overcome negative factors such as personal emotions and fatigue, enabling them to focus on managing every alarm and ensuring patient safety. As N24 said: “Even when I’m really tired on night shifts, the moment the central alarm sounds, I spring into action—it’s an instinctive response.” N5 described a typical case: “I was in charge of Bed 20 last week. Most alarms were false, but at one point, the patient’s blood pressure suddenly dropped. If I had muted the ECG monitor, I might have missed it, and the patient could have died. Even though it’s annoying and I often want to silence the alarms, I believe they should never be turned off.” This scenario represents a classic near miss, where a timely response—enabled by the nurse’s conscious decision not to silence the alarm—prevented a potentially fatal outcome. It highlights how professional responsibility can override fatigue-driven impulses and sustain clinical vigilance. Furthermore, responsibility not only influences nurses’ response speed to alarms but also directly affects the frequency of alarms. N4 explained: “Nurses who lack responsibility may overlook patient care details, which can lead to frequent alarms. As long as the patient is alive, they do not mind if the alarms go off repeatedly.” In contrast, nurses with a strong sense of responsibility are more engaged in their work and pay closer attention to alarm alerts and patient condition changes, ensuring better patient care.

Extensive work experience: Experienced nurses can more quickly identify alarm issues and take effective measures, thereby reducing alarm-related disturbances. N11 described: “Because there can be many reasons for an alarm, experienced nurses can immediately pinpoint the problem.” They also use their accumulated knowledge and skills to better anticipate patient conditions and potential alarms. N21 mentioned: “It’s related to work experience. After mastering specialty knowledge, you can anticipate what kinds of alarms might happen with patients.” This anticipatory ability enhances nurses’ vigilance, enabling early identification of potential risks and timely intervention, thus reducing delays caused by fatigue. Furthermore, experienced nurses can reduce alarm occurrences and alleviate cognitive load by implementing reasonable care plans. N22 stated: “Senior nurses perform comprehensive care during work, such as sedation, suctioning, and alarm parameter settings, which greatly reduces alarms.”

##### 3.2.2.2 Inadequate support resources for alarm response

Lack of practical training: Although theoretical training is systematic and comprehensive, most nurses report that existing training lacks practical application and does not fully address challenges encountered in real clinical settings. For example, N24 stated: “No training has ever told me what the ventilator alarm sounds like.” Another nurse, N6, expressed: “Regarding how to handle alarms, I think we really need more practice. Our current training sometimes feels more like a formality.”

Absence of formal regulations: Almost all nurses report that there are no formal, written regulations for managing alarms in their daily work. Current practice largely relies on informal conventions or experiential teaching within the profession. N16 stated: “Most of the time, senior nurses just give you a brief explanation and they only teach you how to handle issues when problems arise. There is no specific training for this.” Some nurses described existing practice as “an agreement, basically a habitual action.” (N21).

Outdated and malfunctioning equipment: The issue of outdated and malfunctioning equipment significantly increases nurses’ external cognitive load in alarm management. Many nurses report that older devices respond slowly and are difficult to operate, requiring more time and effort, which reduces alarm response efficiency. As N15 described: “Some monitors are quite old; they do not have touch screens, and the adjacent buttons do not work well either. You have to navigate through the interface using knobs to find alarm settings and mute functions, which is very time-consuming.” Such cumbersome operation often leads nurses to ignore alarms. N5 said: “I get so annoyed that I cannot even be bothered to silence it anymore. I just leave it.” Additionally, alarms with unadjustable high volume increase nurses’ emotional stress. N8 said: “Honestly, I just want to smash it. That crazy green pump pierces my eardrums like a sharp knife.” It is worth noting that N15 further reported recurring failures of the aging 840 ventilator: “After a period of use, it alarms automatically and stops supplying gas. I’ve encountered this many times, and each time we have to replace it. This significantly increases our workload. Nurses and respiratory therapists find it frustrating. If the patient has no spontaneous breathing, the situation could become extremely dangerous.” This represents a recurrent near miss, where timely intervention consistently prevents potentially adverse outcomes, but at the cost of increased staff burden, sustained clinical risk, and heightened susceptibility to alarm fatigue.

Crowded and noisy layout: A crowded spatial layout directly affects nurses’ ability to respond to alarms promptly, especially during physician rounds and multidisciplinary activities in the ward. For example, N25 said: “The EICU space is very limited. The ventilator is at the bedside, and sometimes there are hemodialysis machines, tables, and other obstacles beside the bed, making it very difficult to operate the ventilator, which certainly slows down alarm response.” N11 also stated: “When there is no one around, you can quickly respond, but if there are many people, it obstructs your movement.” Moreover, the open bay room design intensifies environmental noise. N16 described: “Our unit has all patients lined up in a row. It’s very noisy.” Nurses generally believe that environmental noise masks equipment alarms and affects alarm recognition. N14 reported: “When the noise is too loud, the alarm sounds become inaudible. All sorts of noises mixed together affect our thinking.” In the open bay room, even alarms from patients not under their care distract nurses’ attention. N12 said: “If one alarm sounds, all other nurses will hear it and look for the source.”

Emotional personality traits: Nurses generally believe that personality traits influence their cognitive responses and decision-making when facing alarms. An impulsive nurse tends to be highly sensitive to alarm sounds, showing higher response efficiency but also more pronounced negative emotions. As N11 described: “Impatient nurses will respond immediately when they hear an alarm, regardless of its urgency.” N10 added: “Short-tempered individuals feel irritated by alarm sounds, whereas nurses with a calm personality remain more composed.”

Insufficient or poor sleep: Insufficient or poor sleep increases nurses’ fatigue, directly impairing their response speed and judgment, while also placing them under heightened emotional stress. Sleep deprivation was commonly reported to slow reaction time. As N17 noted: “When I have not rested well, I need more time to react.” In addition to delayed cognitive responses, sleep deprivation also reduced nurses’ willingness to respond to alarms. N5 explained: “When I’m really sleepy, I feel lazy. I do not want to keep moving or doing things, and I become more annoyed by the alarms.” N15 added: “When I do not sleep well, I tend to feel anxious, and it’s hard to control my emotions.” N3 shared his experience: “Once I was very tired, a heart rate alarm went off, and I just mechanically pressed the mute button. If I had been feeling better, I would have been more proactive—calling the doctor, adjusting alarm parameters, and so on.”

Suboptimal health status: When feeling unwell, nurses often experience reduced alarm sensitivity and slower response times. N12 remarked: “When I have a fever, my attention drops significantly. I just do not have the energy to deal with alarms.” N8 observed: “I react slowly and sometimes cannot tell where the sound is coming from when I’m sick.” Motivation to respond also declines, making nurses more likely to ignore non-urgent alarms. N5 admitted: “When I have a cold, I feel dizzy and do not want to deal with anything. I just let it go.” Even when carrying out their duties, some nurses acknowledged handling alarms in a state of emotional irritation. As N25 put it: “I deal with it, but I feel irritable inside.”

## 4 Discussion

### 4.1 Cognitive load as a mechanism of alarm fatigue

Alarm fatigue depends on nurses’ cognitive load states, which can be categorized into three stages during alarm management: cognitive reserve deficit, cognitive load balance, and cognitive overload, based on the dynamic match between available cognitive resources and task demands. Each stage is characterized by three interrelated dimensions: alarm perception, alarm response, and emotional and psychological reactions. Alarm perception involves auditory detection and cognitive sensitivity, which together influence nurses’ alertness and subsequent behavioral responses (45). Alarm response refers to the concrete actions undertaken by nurses in response to alarm events (46). Emotional and psychological reactions reflect the affective changes experienced by nurses when exposed to alarm-rich environments, which may further manifest as physical discomfort (47).

Cognitive reserve refers to relevant knowledge stored in long-term memory and serves as a foundational resource for nurses when performing alarm-related tasks. Although cognitive reserve deficit may lead to delayed alarm responses, it should not be regarded as alarm fatigue in isolation. At this stage, novice nurses are required to simultaneously process multiple interacting elements in working memory, including patient condition, medical devices, monitoring parameters, and procedural workflows (11), with potential irrelevant environmental distractions also present (48). The time required for this process is not entirely within the control of the individual and should not be interpreted as a subjective “delay” or “desensitization” by nurses. This stage is more accurately described as an adaptation period for novice nurses, as noted by Nusair et al. (49).

The findings indicate that cognitive overload is the critical trigger for alarm fatigue. When the demands of alarm response exceed nurses’ working memory capacity, information elements are lost during processing, and the meanings of the lost information, as well as their relationships with other data, cannot be adequately interpreted (50). This results in impaired alarm perception and simplified judgment. Furthermore, research shows that during cognitive tasks, working memory resources are required not only to meet task demands but also to suppress negative behaviors such as resting or idleness (51, 52). Under cognitive overload, insufficient working memory resources are available to inhibit negative behaviors (53), which consequently leads to inadequate alarm responses.

Previous studies (1, 54) have typically defined alarm fatigue in terms of a decline in alarm response time and response rate; however, the results reveal that it is also accompanied by psychological and emotional changes in nurses. Therefore, this study extends the definition of alarm fatigue as (55): (i) triggered by the cognitive, physical, and emotional effort exerted in alarm management, (ii) accompanied by unpleasant subjective experiences, such as irritability or numbness, and (iii) manifested as reluctance to respond to alarms, leading to impaired perception and inadequate responses. This definition is grounded in the three-stage mechanism proposed in this study, which links alarm fatigue to nurses’ cognitive load states. Fatigue-related symptoms are most likely to emerge during the cognitive overload stage, where task demands critically exceed available support resources, leading to behavioral disengagement and emotional or psychological exhaustion.

### 4.2 Factors contributing to alarm fatigue

Alarm fatigue can be both chronic and cumulative, while also being dynamically shaped by the interaction of multiple factors (56, 57). Whether nurses transition from an initial state of cognitive reserve deficit to a balanced or overloaded cognitive state depends on the intensity of alarm response demands and the availability of supportive resources (Figure 3).

#### 4.2.1 Alarm response demands: the source of cognitive load

In the ICU, nurses face heavy workloads (58), with various demands intertwined that impose multiple pressures on their working memory.

First, the high frequency of alarms is a major source of cognitive load. A study has reported that ICU alarm load averages 152.5 ± 42.2 alarms per bed per day (59). Each time an alarm sounds, nurses must rapidly shift attention, prioritize tasks, and reallocate their limited working memory resources (60). This frequent task switching and continuous information processing increase nurses’ cognitive load and deplete working memory resources (61, 62), progressively raising the risk of alarm fatigue. Secondly, repeated false alarms and other non-alarm nursing tasks increase the number of interacting elements in nurses’ working memory, adding to the external load of alarm management (37). False alarms create unnecessary distractions and a “cry wolf” effect, causing nurses to ignore or underestimate alarm threats (63, 64). When other nursing tasks occur during alarm processing (and vice versa), limited working memory resources are divided (65), reducing task efficiency and accuracy (66). Furthermore, this study found that although the quieter night shift environment reduces external noise-related load, nurses are more sensitive to alarm sounds but show slower reaction times and less standardized care practices, consistent with research indicating impaired cognitive performance during night shifts (67, 68).

In addition to direct response demands, work–family conflict also contributes to nurses’ external load. Studies have shown that nurses frequently experience work–family conflict, which is closely associated with lower care quality indices and higher turnover intention (69, 70). According to the findings of this study, such conflict impairs attention control and disrupts the allocation of working memory resources (66), leading to delayed and inappropriate alarm responses.

#### 4.2.2 Alarm response resources: modulating factors of cognitive load

Alarm response resources help reduce both intrinsic and extraneous loads that nurses face during alarm tasks, while simultaneously increasing germane load, thereby playing a crucial role in alleviating alarm fatigue. However, when resources are inadequate, cognitive load increases, leaving nurses feeling overwhelmed and exacerbating feelings of fatigue.

##### 4.2.2.1 Effective support resources: alleviating cognitive load

Supportive job resources primarily include effective team collaboration, management’s emphasis on alarm issues, and comprehensive theoretical training. When nurses perform nursing tasks independently, all the interacting elements involved must be processed within their single working memory. In contrast, in a team environment, these interacting elements can be distributed across multiple working memories (i.e., those of different nurses), effectively reducing the cognitive load on any single nurse (71). Therefore, effective team collaboration can serve as a protective factor against alarm fatigue for nurses (64). In most cases, performance on cognitive tasks declines over time (72). However, management’s emphasis can enhance nurses’ motivation to respond to alarms, reversing this decline (73). Organizational training can rapidly increase nurses’ knowledge reserves, thereby boosting their germane load when handling clinical alarm tasks (74).

Supportive personal resources primarily include nurses’ strong psychological adaptability, a strong sense of responsibility, and extensive work experience. Psychological adaptability reflects the capacity for self-regulation under the high demands of ICU work (75). Nurses with high adaptability can cognitively reframe stressors, accommodate the high-intensity demands for alarm response, and mitigate the effects of emotional dissonance (76, 77). A strong sense of responsibility enhances motivation to engage in cognitive tasks, as care quality is often perceived to be closely linked to patient safety. Therefore, nurses tend to remain proactive in managing alarms, even under adverse conditions (78). Work experience significantly influences germane cognitive load in alarm management. Compared to novice nurses, experienced nurses have accumulated a richer cognitive reserve through long-term practice and have developed internalized schemas for alarm management. These schemas allow them to bypass step-by-step deliberation and directly identify meaningful patterns or priorities within complex alarm situations (48). As a result, they can process alarm information more holistically and efficiently, with greater foresight and planning, and with less cognitive strain during task execution.

##### 4.2.2.2 Inadequate support resources: increasing cognitive load

Currently, inadequate job resources for alarm management in clinical settings include a lack of practical training, the absence of formal regulations, outdated or malfunctioning equipment, crowded and noisy layout. First, lack of practical training hinders the effective transfer of theoretical knowledge to alarm-handling scenarios (38). Nurses may struggle with alarm recognition and response procedures, limiting improvements in germane cognitive load. Second, formal regulations are essential for reducing cognitive demands. According to cognitive load theory, clearly structured tasks and explicit guidance allow individuals to respond based on established rules, thereby minimizing working memory usage (50). The absence of such regulations increases uncertainty and decision-making burden (64), and hinders the consolidation of response procedures into long-term memory (48), ultimately reducing alarm response efficiency. Finally, equipment and environmental conditions—key external supportive resources—directly influence extraneous cognitive load. Outdated and malfunctioning equipment, limited workspace, and high noise levels increase cognitive effort and elevate the risk of alarm fatigue (79).

Inadequate personal resources for alarm response include emotional personality traits, insufficient or poor sleep, and suboptimal health status. According to the job demands-resources model, personality traits shape how individuals perceive job demands and available resources (32). In this study, nurses with irritable temperaments perceived alarms as more stressful, leading to greater emotional fluctuation, increased extraneous cognitive load, and a higher risk of fatigue (80, 81). Sleep quality (82) and general health (83) are critical physiological factors contributing to alarm fatigue. When in poor physical condition, nurses tend to exhibit lower motivation and slower cognitive processing, thereby impairing the effectiveness of alarm response (51, 79).

### 4.3 Strategies for managing alarm fatigue

Preventing and alleviating alarm fatigue is essential not only for nurses’ well-being but also for ensuring care quality and patient safety (84). Based on interview findings, effective strategies should target two key areas: reducing demands and enhancing resources for alarm response, thereby alleviating nurses’ cognitive load (85).

At the organizational level, healthcare institutions are encouraged to implement system-based intelligent interventions—such as alarm integration (86), filtering (87), dynamic threshold setting (88), and multisensory alarm delivery (89)—to improve alarm accuracy and reduce their overall frequency. These measures are critical for minimizing unnecessary alarm load and task interference, thereby alleviating nurses’ cognitive load and emotional burden (90). Building on this foundation, emerging technologies such as remote monitoring and artificial intelligence (AI) provide new avenues for managing alarm fatigue and improving safety. For example, alarm signals from multiple wards can be transmitted in real time to a centralized command center, where trained personnel conduct preliminary screening and analysis (91). This reduces unnecessary on-site interventions and mitigates the risks of overreliance on individual bedside nurses. Moreover, automated alarm reasoning (92), classification (93), and prioritization mechanisms (94) ensure that high-risk alerts are addressed first during multitasking, thereby minimizing delays due to subjective decision-making. AI algorithms can detect abnormal events, predict clinical deterioration, and offer real-time decision support, shifting alarm management from passive reaction to proactive recognition and prediction (95, 96). These technologies enable cross-temporal and cross-spatial coordination, streamline alarm response workflows, and extend the functional scope of traditional alarm systems. Collectively, they show strong potential to reduce cognitive load and improve operational efficiency.

Such strategies have been extensively validated in the aviation industry. In the early 20th century, aircraft required multiple crew members to monitor various sensors and displays (97). Today’s cockpits are highly automated, allowing pilots to manage most cognitive tasks independently (98). Alarms are categorized by priority and mostly presented visually to reduce disruption, which contrasts sharply with the frequent and often trivial audible alarms in ICUs (94). Unlike standardized mechanical systems, the physiological status of critically ill patients is highly dynamic, influenced by diagnostic, clinical, and comorbid factors, making it challenging to define fixed safe thresholds (99). Nevertheless, core aviation alarm principles provide valuable guidance for ICU alarm safety and design: prioritizing critical information, minimizing irrelevant alarms, maintaining situational awareness, and preventing information overload (100).

Furthermore, organizations must prioritize reasonable shift scheduling and ensure adequate rest breaks, as rest periods have been shown to facilitate the restoration of working memory resources (101). Establishing a flexible backup staffing system to accommodate nurses’ leave for illness or work–family conflict is essential for promoting a supportive work environment. Collectively, these institutional actions sustain nurses’ cognitive engagement during shifts and support effective alarm response and high-quality patient care (102, 103).

Nursing managers should emphasize alarm response by establishing clear regulations and providing explicit guidance to help nurses consolidate coping strategies into long-term memory schemas, thereby improving adherence and alarm handling efficiency (104). A parallel can be drawn from aviation, where alarms are managed through structured response protocols: critical alarms are silenced after identification to reduce overload, and pilots follow standardized checklists and team-based frameworks such as T-FORDEC to guide decisions under pressure (105). Similarly, nursing managers should implement continuous monitoring and feedback systems that promptly identify problems and recognize exemplary performance, facilitating nurses to internalize high-quality alarm response and translate it into consistent practice. Regular departmental training should be systematically and rigorously conducted, combining theoretical instruction with practical exercises to build nurses’ cognitive reserves and reduce reliance on experience. Training programs must also include relaxation techniques to enhance nurses’ psychological resilience under high workload. In addition, routine maintenance and upgrading of equipment (106), along with optimizing environmental layout (e.g., noise reduction) (107), are essential to decrease extraneous cognitive load and improve the timeliness of alarm responses.

At the team level, fostering a culture of accountability and professionalism can gradually strengthen nurses’ professional identity and sense of responsibility. Enhancing team collaboration and support helps distribute nurses’ cognitive load more evenly, easing the pressure caused by high-intensity alarm demands (90). Additionally, for nurses who are emotionally vulnerable, timely care and support from team members are essential to help them effectively cope with alarm tasks and reduce emotional exhaustion.

At the individual level, nurses should recognize the importance of timely alarm response, cultivate a strong sense of responsibility, and maintain a calm and composed attitude at work. They are encouraged to embrace lifelong learning, actively build their cognitive reserves, and continuously expand their professional knowledge and clinical experience to enhance alarm management efficiency and adaptability. When experiencing emotional stress or physical discomfort, seeking timely support can help prevent alarm fatigue and related risks. In daily life, adopting a healthy lifestyle—such as engaging in brief physical exercises during breaks—may improve physical fitness and sleep quality, reduce fatigue and stress, and support the restoration of working memory resources (108).

### 4.4 Strengths and limitations

This study is the first to explore alarm fatigue from the perspectives of cognitive load and job demands-resources model, thereby deepening the theoretical understanding of this phenomenon. By selecting three hospitals of different sizes, various ICU types, and nurses with diverse experience, a wide range of perspectives was captured, improving the credibility of the findings.

However, limitations exist. This study focuses exclusively on ICUs, which limits the generalizability of its findings. Compared to ICUs, patient conditions in operating rooms change more rapidly due to anesthesia fluctuations and surgical events such as massive hemorrhage (109). As a result, the alarm burden in operating rooms is often more intense and time-sensitive. Studies report an average of 359 ± 158 alarms per procedure (110), with over 70% classified as technical or clinically non-actionable alarms (111). Nurses and anesthesia professionals in operating also experience alarm fatigue (112, 113). Further research in these settings is essential to develop a more comprehensive understanding of alarm fatigue across clinical environments. Additionally, due to the sensitive nature of the topic, data rely on self-report and may be influenced by social desirability bias (114). Participants may have unintentionally avoided reporting their own inappropriate behaviors in alarm responses, instead describing issues related to others. While the study reveals that alarm fatigue changes in response to the balance between available resources and alarm demands, the cross-sectional nature of the data limits our ability to fully capture how this balance evolves. Nonetheless, the consistent emergence of key points supports the reliability and validity of the results.

## 5 Conclusion

Alarm fatigue is closely linked to the cognitive load experienced by ICU nurses, which acts as the underlying mechanism of its development. This cognitive load results from the dynamic balance between alarm response demands and available resources. When resources are inadequate to meet the high demands of alarm response, nurses experience cognitive overload, leading directly to alarm fatigue. Addressing alarm fatigue requires reducing alarm-related demands and enhancing supportive resources through coordinated actions at organizational, team, and individual levels. Each ICU unit should develop targeted measures based on its specific context to improve nurse well-being, ensure patient safety, and enhance the quality of critical care. Future research employing a longitudinal design could provide deeper insights into the dynamic nature of the balance between alarm demands and resources, offering a more comprehensive understanding of the development and impact of alarm fatigue.

## Data Availability

The raw data supporting the conclusions of this article will be made available by the authors, without undue reservation.
