# Supplementary material for: Mechanism, contributing factors, and coping strategies of alarm fatigue in intensive care nursing: a qualitative study
Source: Front Public Health. 2025 Oct 3;13:1654389. doi: 10.3389/fpubh.2025.1654389 (PMC12531151; doi:10.3389/fpubh.2025.1654389)
Supplement: Supplementary file 1 [file Data_Sheet_1.docx]

Appendix A: Outline of the semi-structured interview guide on alarm fatigue

| Questions |
| --- |
| 1. How do you typically feel or react when alarms sound in the ICU? |
| 1. Has your response to alarms changed over time? If so, can you describe any differences you have noticed? |
| 1. Have you ever chosen to ignore or silence an alarm? If so, in what situations does this usually occur? |
| 1. Do you think you or those around you have ever experienced alarm fatigue? If so, could you describe what that experience was like? |
| 1. In your opinion, what factors may contribute to alarm fatigue? |
| 1. What factors do you think could help prevent alarm fatigue? |
