# Supplementary material for: Mechanism, contributing factors, and coping strategies of alarm fatigue in intensive care nursing: a qualitative study
Source: Front Public Health. 2025 Oct 3;13:1654389. doi: 10.3389/fpubh.2025.1654389 (PMC12531151; doi:10.3389/fpubh.2025.1654389)
Supplement: Supplementary file 2 [file Data_Sheet_2.docx]

**Appendix B: Categories and representative quotes of influencing factors on alarm fatigue**

| Theme | Category | Sub-category | Codes | Representative quotes |
| --- | --- | --- | --- | --- |
| Influencing factors of alarm fatigue | High-intensity demands for alarm response | NA | High alarm frequency | “The alarms go off all the time... they basically never stop, especially when you’re resuscitating a patient—you end up listening to the heart monitor alarms all night.” (N12) |
|  |  |  | Persistent false alarms | “When the false alarms keep going off nonstop for so long, I honestly just feel like turning the monitor sound off altogether and watching the patient myself. But then I worry—what if something actually happens? So I don’t dare. In the end, you just start to feel numb.” (N5) |
|  |  |  | Multitasking | “When things get really busy and the alarms start going off again, I get irritated... it just makes everything feel even more overwhelming.” (N26) |
|  |  |  | Night shifts | “On night shifts, you’re tired and groggy, and your reactions just slow down.” (N10) |
|  |  |  | Work-family conflict | “Those of us in our 30s with young kids are often sleep-deprived from looking after them, and that exhaustion inevitably spills over into work.” (N14) |
|  | Multilevel resources for alarm response | Effective support resources for alarm response | Effective team collaboration | “The ICU is definitely busy, but the team atmosphere is great. For instance, when I’m overwhelmed and a coworker steps in to deal with an alarm, it makes my work so much easier and really lightens how I feel.” (N23) |
|  |  |  | Management’s emphasis | “When the management takes monitor or ventilator alarms seriously, it makes us pay more attention to them as well.” (N9) |
|  |  |  | Comprehensive theoretical training | “We have relevant training, like how to adjust the parameters on the monitors.” (N25) |
|  |  |  | Strong psychological adaptability | “I think it’s really about your own mental adjustment. I’ve always felt that when I face a problem, I can gradually adapt to it and find a way to solve it.” (N6) |
|  |  |  | Strong sense of responsibility | “I think you should have a strong sense of responsibility at work. We’re talking about lives here—each one a living, breathing person. What could be more important than that?” (N11) |
|  |  |  | Extensive work experience | “I’ve been in the ICU for over ten years... managing alarms is really easy for me now.” (N4) |
|  |  | Inadequate support resources for alarm response | Lack of practical training | “Whenever I ran into an alarm issue, I would ask more experienced colleagues how to handle it, and that’s how I learned.” (N1) |
|  |  |  | Absence of formal regulations | “There aren’t any specific rules or regulations for alarm management. It’s all just something we’ve agreed on verbally.” (N12) |
|  |  |  | Outdated and malfunctioning equipment | “When you adjust the infusion pump’s speed, it takes ages for it to respond.” (N25) |
|  |  |  | Crowded and noisy layout | “Our ward’s just one big open area, super noisy... with so many machines, you can’t quickly figure out which one’s alarming.” (N16) |
|  |  |  | Emotional personality traits | “Some nurses are naturally cheerful and lively, so they face alarms optimistically. But others aren’t like that—they start feeling anxious as soon as they get to work.” (N15) |
|  |  |  | Insufficient or poor sleep | “Some nurses don’t sleep well, which makes them tired at work. For instance, they might barely notice a blood pressure alarm.” (N20) |
|  |  |  | Suboptimal health status | “I was feeling really unwell, but I came to work that night anyway. When alarms went off, I just didn’t have the energy to respond. I had a trainee with me, and I ended up letting her take care of some alarms. Working while sick made me feel like nothing mattered more than my own health.” (N6) |

Note: **NA** is used to indicate that high-intensity demands for alarm response does not have a corresponding sub-category.
